# Plant Population and Row Spacing Affects Growth and Yield of Rainfed Maize in Semi-arid Environments

**DOI:** 10.3389/fpls.2022.761121

**Published:** 2022-06-08

**Authors:** Stephanus J. Haarhoff, Pieter A. Swanepoel

**Affiliations:** Department of Agronomy, Faculty of AgriSciences, Stellenbosch University, Stellenbosch, South Africa

**Keywords:** leaf area index, conservation agricultural practices, soil water, dryland agriculture, regenerative agriculture, corn, row width, plant density

## Abstract

Increased tolerance to competition for soil resources of modern maize (*Zea mays* L.) hybrids increases soil resource use efficiency and yield. Yet little information is available on the relationship between maize population density and yield under no-tillage in semi-arid environments. A 2-year field trial was conducted in South Africa during the 2017/2018 (Season 1) and 2018/2019 (Season 2) production seasons to evaluate growth and water use productivity of rainfed maize established at seven diverse plant population (20,000–60,000 plants ha^−1^) and row spacing (0.52 and 0.76 m) configurations. In Season 1, light interception was 6.8% greater at 0.76 m row spacing compared to 0.52 m row spacing (*p* < 0.05). In Season 2, despite dry and hot growing conditions, a well-developed leaf canopy cover was present at 0.52 m row spacing indicating a 10.4% greater intercepted photosynthetically active radiation (IPAR) compared to 0.76 m row spacing. In Season 1, with more uniform rainfall distribution, no biomass or yield benefits were found with increased plant population, except at 50,000 plants ha^−1^ at 0.76 m row spacing. In Season 2, plant populations at 0.76 m row spacing out-yielded any given plant population at 0.52 m row spacing. The optimal plant population and row spacing will ultimately be a compromise between obtaining high maize grain yield and minimizing the potential for crop failure in semi-arid environments.

## Introduction

Maize (*Zea mays* L.) produced under rainfed conditions is among the most important crops in semi-arid environments in various regions in the world, including parts of the United States, Northeast China, and South Africa (Clay et al., 2014; Qin et al., 2016; Haarhoff and Swanepoel, 2020). Semi-arid environments are characterized by high summer day temperatures and low or inconsistent rainfall where lengthy dry spells commonly occur during the growing season (Zuma-Netshiukhwi et al., 2013). As a result, the evapotranspiration greatly exceeds rainfall in semi-arid environments. For example, across the semi-arid maize production region of South Africa, evapotranspiration may exceed 2,500 mm *per annum* (Walker and Schulze, 2008), while long-term annual rainfall ranges between 400 and 550 mm. This disparity between rainfall and evapotranspiration highlights the importance of utilizing the available soil resources, particularly plant-available water, effectively (Haarhoff et al., 2020).

Improved agronomic practices often lead to improved efficiency of maize production (Swanepoel, 2021). Modern weed and pest management practices (Teasdale, 1998), crop residue retention (Sindelar et al., 2013), and soil tillage management strategies (Perez-Bidegain et al., 2007), provide pathways to reduce the effect of drought conditions on yield. Genetic advances coupled with increased plant population were major factors explaining recent maize grain yield improvements (Duvick, 2005). Modern hybrids are more stress-resilient and can withstand greater interplant competition enabling producers to increase maize grain yields through increasing the number of plants per unit area in more humid environments (Ma et al., 2014). Yield benefits of narrow row spacing depend on increased radiation interception (Andrade et al., 1999), which is generally accompanied by a reduction in evaporation from the soil surface. Additional benefits include a more uniform crop root distribution (Hammer et al., 2009) and improved weed control strategies (Sardana et al., 2017). Previous studies indicated that weed growth and nutrient uptake by weeds were significantly reduced when increased maize plant populations coupled with narrower row spacing was established (Arvadiya et al., 2012; Jha et al., 2017). Early leaf canopy closure and the greater shading of weeds results in an increase in the competitive ability of the growing crop (Singh et al., 2013). Promoting more efficient uptake and use of available soil water and nutrients (Sandler et al., 2015) using greater plant densities is critical in semi-arid environments for achieving sustainable yields under rainfed conditions (Haarhoff and Swanepoel, 2018). Despite these benefits, producers in semi-arid environments still opt for low plant populations (<30,000 plants ha^−1^) established at a wide row spacing (> 0.91 m) to minimize risk of crop failure. Therefore, there exists a need to re-evaluate plant population and row spacing configurations under newly introduced agronomic practices to improve crop performance in these drier environments while still preserving the farmers’ needs to minimize risk (Haarhoff et al., 2020).

Functional processes may depend on site and season characteristics (environment), such as soil water availability (Nielsen et al., 2010), soil water content at planting, rainfall amount and distribution, and the interaction between these characteristics with management practices. For example, plant population and row spacing determine the onset of competition between plants for resources and different biomass production (Tetio-Kagho and Gardner, 1988). Ample soil water during early vegetative growth stages may promote leaf expansion (thereby increasing radiation interception) and lead to excessive biomass production. When a prolonged dry spell occurs later in the growing season, a high leaf area index (LAI) promotes soil water extraction, resulting in a dry soil during the critical period for kernel set, hence severely affecting grain production.

The success of increased plant population and/or narrow row spacing is well-known in wet and humid environments such as in the United States Corn Belt (Duvick, 2005), Southwestern China (Qin et al., 2016), and the Argentine Pampas (Echarte et al., 2000). A comprehensive systematic review revealed that despite the increasing number of studies performed globally on plant population and row spacing, less than 5% were performed under no-tillage in semi-arid environments (Haarhoff and Swanepoel, 2018). Therefore, to fill this gap in information for these environments, rainfed field trials were conducted in the semi-arid maize production region of South Africa to evaluate the effects of maize plant population and row spacing on (i) aboveground growth and development; (ii) soil water-use productivity; and (iii) grain yield and yield components under no-tillage.

## Materials and Methods

### Site Description

Field trials were conducted near Ottosdal (26°47′ S, 25°56′ E; altitude 1,490 m), North-West Province, South Africa, during the 2017/2018 (Season 1) and 2018/2019 (Season 2) production seasons. The region has a semi-arid climate (BSk) with a mean annual rainfall of 447 mm (Kottek et al., 2006). Approximately 90% of the annual rainfall occurs in the summer growing season (October to April). Rainfall patterns are highly inconsistent between seasons and dry spells during the growing season are common phenomena.

Soil type is a hard-xanthic Plinthic Haplustox (Soil Survey Staff, 2003). Soil bulk density in the 0–60 cm soil depth was 1.6 g cm^−3^ at the onset of the trial in Season 1. Soil texture was sandy loam with organic matter content of 0.9%. The experimental site has been under no-tillage since 2011. Maize monoculture practices were followed in the field trial and soil cover was approximately 95% in the 2 months following harvest. Strong winds during winter removed a large portion of the crop residues resulting in a soil cover of 35%–40% on the day of planting in each season. Maize monoculture is a common practice across the summer grain production region of South Africa due to favorable markets and livestock feed needs during the winter months (Haarhoff et al., 2020).

Cumulative growing degree days (GDD) were calculated according to Gilmore and Rogers (1958) using daily air temperature data provided by the South African Weather Service. The GDD base temperature was set as 10°C. Air temperature was measured at a weather station approximately 10 km from the trial site. Rainfall was recorded at the trial site using a manual rain gage.

### Field Trial Design and Treatments

The experimental design was a randomized split-plot design with four blocked replicates. Whole-plots were row spacing (0.52 and 0.76 m), while plant population formed sub-plots, randomly nested within whole-plots (Table 1). These plant population configurations were chosen to achieve similar intra-row spacings in each row spacing as practiced by local producers. Plant populations of between 15,000 and 28,000 plants ha^−1^ are currently established by rainfed producers under conventional tillage conditions across the local region.

**Table 1:** Plant population and row spacing configurations and resultant intra-row spacings between plants as treatments.

Row spacing (m)			
0.52		0.76	
Plant population (plants ha^−1^)	Intra-row spacing (cm)	Plant population (plants ha^−1^)	Intra-row spacing (cm)
25,000	76	20,000	66
38,000	48	30,000	44
50,000	38	40,000	33
60,000	32	50,000	26

Plot lengths were 20 m and plot width depended on row spacing. Plots with 0.52 m row spacing had 12 rows leading to plot widths of 6.2 m, while the 0.76 m row spacing plots had 10 rows leading to 7.6 m widths. Plots were overplanted at 65, 000 plants ha^−1^ to ensure a high stand, and hand-thinned to the target plant populations at the fifth-leaf collar (V5) development stage (Ritchie et al., 1993), leaving a stand with uniform intra-row spacing in each treatment. The plots used in Season 1 were also used in Season 2 to include compounding effects of root biomass accumulation in the soil from the use of different plant densities. Maize plant density prior to Season 1 was 25, 000 plants ha^−1^.

### Trial Management and Calculations

Representative soil samples were taken prior to planting to establish baseline chemical properties. In both seasons, nitrogen was broadcasted prior to planting as urea at 75 kg N ha^−1^, while 14 kg N ha^−1^ was band-placed as monoammonium phosphate at planting. Maize was planted by means of direct-drilling, using a 10-row John Deere 2117 no-tillage planter (John Deere Pty (Ltd.), Iowa, United States) and a six-row Jumil 2670-EX POP no-tillage planter [Jumil, Pty (Ltd.), Castelo, Espírito Santo, Brazil] in the 0.76 and 0.52 m row spacing plots, respectively.

The trials were established on 14 December 2017 and 4 January 2019 in Season 1 and 2, respectively. The optimal planting window for achieving maximum maize grain yield potential in the North-West province ranges between mid-November to mid-December. Early-autumn frost may occur at the end of April during kernel filling resulting in complete crop loss. Due to very hot conditions and low rainfall at the onset of Season 2, as recommended to farmers, planting was delayed beyond these dates. The 120-day Pioneer maize hybrid P2864WBR was used in both seasons (DuPont Pioneer Hi-Bred International). This hybrid was selected because it is one of the highest yielding cultivars in the region and commonly planted by local rainfed maize producers. Weeds were chemically controlled with pre-emergence herbicides after planting. Although weed pressure was low, hand-weeding was done throughout the growing seasons if necessary to keep plots weed free.

Total biomass was evaluated after emergence by randomly selecting five plants in each plot at 30, 60, 90, and 120 days after emergence (DAE). At least, 75% of plants reached the sixth-leaf collar (V6) stage at 30 DAE, tasseling (VT) at 60 DAE, the linear development phase of kernel filling (R3–R4) at 90 DAE, and physiological maturity (R5–R6) development stage at 120 DAE. Biomass samples were oven-dried at 60°C for 72 h to remove all moisture.

Intercepted photosynthetically active radiation (IPAR) and LAI were measured at VT, when maximum LAI was achieved, using an LP-80 AccuPAR ceptometer (Decagon Devices Inc., 2017). The 84 cm long probe was placed diagonally across two crop rows, with the two ends of the probe located in adjacent crop rows. This measuring regime is advised for row crops, as it provides a representative sample of the entire PAR environment below and between crop rows. The AccuPAR ceptometer calculates LAI based on the above and below-canopy measurements along with additional variables that relate to the canopy architecture and position of the sun. The IPAR and LAI measurements were done at five random spots within each plot above the leaf canopy (reference measurement, Qa) and at ground level (below-canopy measurement, Qb) between 12:00 and 14:00 on clear and windless days. The IPAR is reported as a percentage and was calculated using Equation 1:


(1) $$IPAR\;\left(\%\right)=\left[1-\frac{Qb}{Qa}\right]x\;100$$

Soil water content was monitored at 2- to 3-week intervals in Seasons 1 and 2 from planting until R5-R6. One galvanized access tube (length 120 cm, diameter 4 cm) was in-stalled per plot using a hand auger (diameter 4 cm) immediately after planting in the middle of two crop rows. A neutron probe (503DR Elite Hydroprobe Model, CPN Inc., Concord, CA, United States) was used to record soil water content at 30, 60, 90, and 120 cm soil depths. To calibrate soil water data, gravimetric soil samples were taken approximately 100 cm from the access tubes at planting (at the same time as the neutron probe readings) using a hand auger (diameter 7 cm) at soil layers 0–30, 30–60, 60–90, and 90–120 cm to determine gravimetric soil water content using the standard gravimetric method (Schmugge et al., 1980). The soil samples were oven-dried for 72 h at 105°C to remove all water. The gravimetric soil water content of each soil sample was converted to volumetric water content by multiplying by the soil bulk density. A linear regression of calibration readings against volumetric water values was calculated and used to calculate volumetric water content from the growing season soil water readings. Volumetric soil water content (cm^−3^ cm^−3^) was then converted to soil water content (mm) per layer by multiplying the volumetric soil water content by the soil layer depth (mm). Crop evapotranspiration (crop ET) was calculated as rainfall minus the change in soil water content (accumulated 0–120 cm soil depth) between subsequent measurements, minus drainage. Runoff was considered negligible as the experimental site is flat (<0.5% slope) and well drained. Water productivity for grain (WPg) and biomass production (WPb) were estimated by dividing maize grain yield and total biomass at R5–R6 by the seasonal crop ET (Hatfield and Dold, 2019).

Maize grain yield was determined by hand harvesting the full length of the center eight and six rows of the 0.52 and 0.76 m plots, respectively. Yield components were determined by randomly selecting 10 plants per plot at harvest. Grain samples were oven-dried at 60°C until constant weight and kernel weight was calculated by weighing a sample of 1,000 kernels. Harvest index was calculated by dividing maize grain yield by biomass as determined at R5–R6. All grain yield data were standardized to a moisture level of 12.5%.

### Statistical Analyses

Statistical analyses were performed by using Statistica version 13.5.0.17 (TIBCO Software, 2018). The Restricted Maximum Likelihood (REML) procedure was used to analyze according to the split-plot design. Three treatment factors were specified as fixed effects, i.e., plant population, row spacing and season, as well as the interaction between all three factors. Block, the interaction between block and plant population and block and row spacing were specified as random terms. The REML procedure was followed because the random factors of the dependent variables are also estimated, which allowed the evaluation of the effects of both row spacing and plant population as well as the interactions, despite dissimilar plant population treatments between the 0.52 and 0.76 m row spacings. Fisher’s least significant differences (LSD) test were conducted at a 5% significance level to determine whether interactions among the three factors of interest were significant. The Bonferroni correction test was used as validation of the Fisher’s LSD test to reduce the chances of obtaining false-positive results (type I errors), since multiple pairwise tests were performed on a single set of data. Normality of residuals and homogeneity of variances were tested and fulfilled the assumptions of the statistical model.

### Growing Conditions

**Figure 1:**
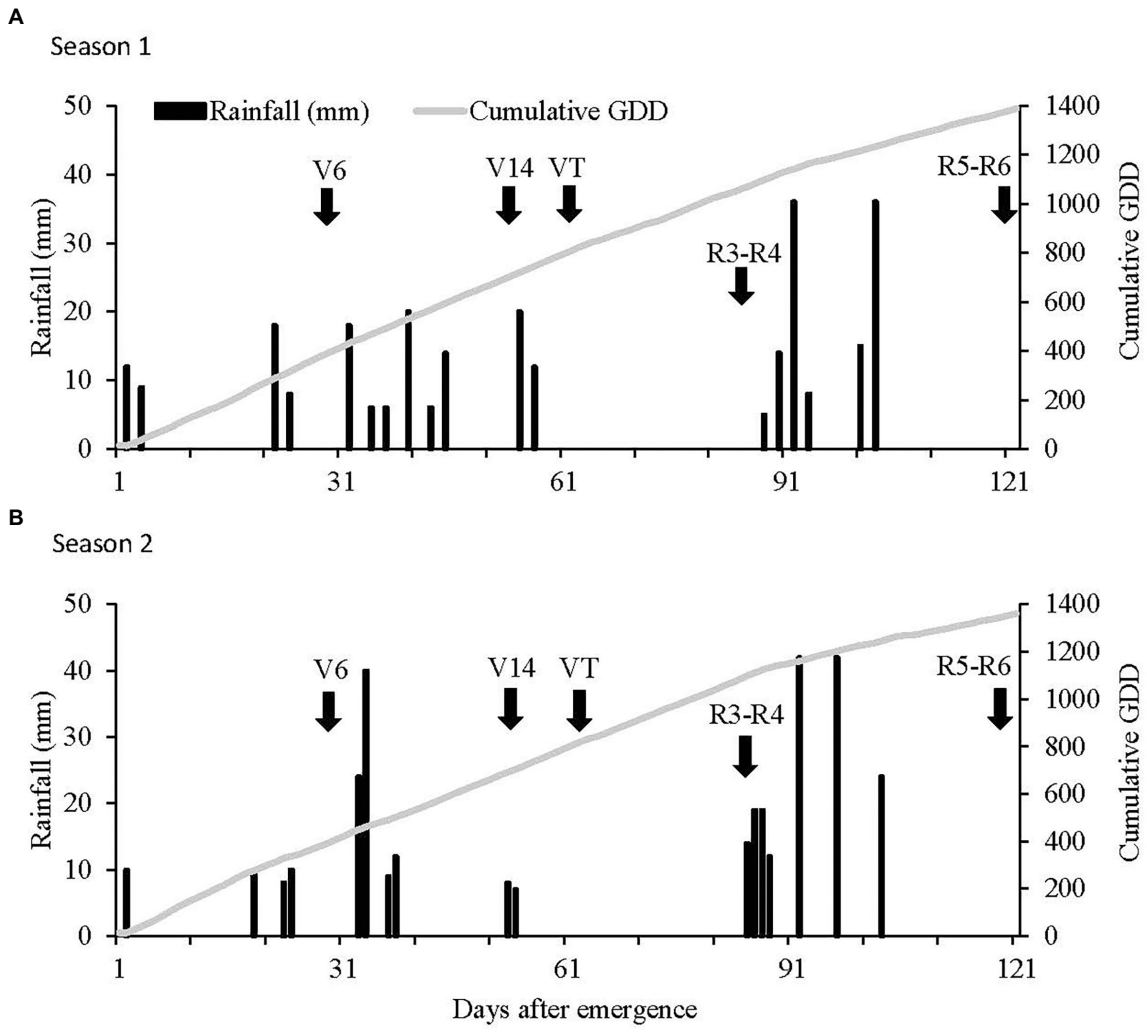
Rainfall events and cumulative growing degree days (GDD) from 0 to 120 days after emergence (DAE) during **(A)** Season 1 and **(B)** Season 2 at the trial site near Ottosdal, South Africa. V6 = sixth-leaf collar, V14 = fourteenth-leaf collar, R3–R4 = the linear development phase of kernel filling, and R5–R6 = physiological maturity.

The total amount of rainfall during the growing period of Seasons 1 and 2 were 263 and 310 mm, respectively. The amount of rainfall for the 8 weeks prior to planting of trials in Seasons 1 and 2 were 83 and 62 mm, respectively. The distribution of rainfall during the cropping seasons was variable and dry spells occurred in both seasons (Figures 1A,B). Despite the late planting date in Season 2, average air temperature was comparable between seasons with cumulative growing degree days (GDD) totaling 1,404 and 1,386 from seedling emergence (VE) to R5–R6 in Season 1 and 2, respectively.

In Season 1, the total amount of rainfall from VE to 14-leaf collar (V14) was 149 mm, corresponding to a deficit of 70 mm compared to the 30-year average. In spite of the low rainfall during this period, soil water status was adequate and early vegetative growth was not affected by the prevailing growing conditions. A dry spell occurred from 57 to 88 DAE when plants were in the early reproductive development stages (VT to R3–R4). Maize plants across all treatments were under severe water stress, thereby negatively affecting kernel development. From 88 DAE onward, wet conditions prevailed with 102 mm received between R3–R4 and R5–R6 allowing satisfactory kernel filling.

Season 2 was characterized by challenging growing conditions from the onset of the season. Between VE and V14, a total of 138 mm of rainfall was received, with only two rainfall events recording more than 15 mm. Between V10 and R3–R4, a prolonged dry spell combined with high air temperatures occurred. Only 15 mm of rainfall was received between the V10 and R3–R4. At this point in the growing season, rainfall received was 130 mm below the 30-year average. Water-stress conditions negatively affected final vegetative growth, pollination, and ear growth across all treatments. Wet conditions and cool air temperatures characterized the period between R3–R4 and R5–R6, allowing maize plants to conclude the latter stages of kernel filling under stress-free growing conditions.

## Results

### IPAR and LAI

Both IPAR and LAI were affected by the interaction between row spacing and season (*p* < 0.05). In Season 1, IPAR was 6.8% greater at the 0.76 m row spacing compared to the 0.52 m row spacing (Table 2; *p* < 0.05). In Season 2, despite challenging growing conditions, a well-developed leaf canopy cover was present at 0.52 m row spacing indicating a 10.4% greater IPAR compared to the 0.76 m row spacing. No differences in LAI were observed between row spacings in Season 1 (*p* > 0.05), however, LAI was 21.8% greater at the 0.52 m row spacing compared to 0.76 m row spacing in Season 2 (*p* < 0.05).

**Table 2:** Effect of row spacing on IPAR and leaf area index (LAI) at tasseling (VT) across all plant populations in Seasons 1 and 2.

Season	Row spacing (m)	IPAR (%)	LAI
Season 1	0.52	74.88^b^	3.75^bc^
	0.76	80.31^a^	4.01^ab^
Season 2	0.52	82.46^a^	4.36^a^
	0.76	73.92^b^	3.41^c^

No common letter indicates a significant difference at level *p* < 0.05 in the ANOVA.

### Soil Water Content and Crop ET

The water content of the soil profile varied over the seasons as a result of variable crop uptake, evaporation rate, and rainfall occurrence (Supplementary Table S1). The soil water content varied with time due to variable plant uptake, evaporation rate, and seasonal rainfall occurrence. Soil water contents were similar at the start and end of the growing season between plant population treatments, irrespective of the row spacing. Also, the timing of water loss from the soil was similar between treatments throughout the growing season. This indicates evaporation at low plant population (< 38,000 plants ha^−1^) due to poor leaf canopy was similar to the evapotranspiration rate at higher plant populations. Crop ET was only affected by the main effect of season and was 255 and 333 mm in Season 1 and 2, respectively (results not shown; *p* < 0.05).

**Figure 2:**
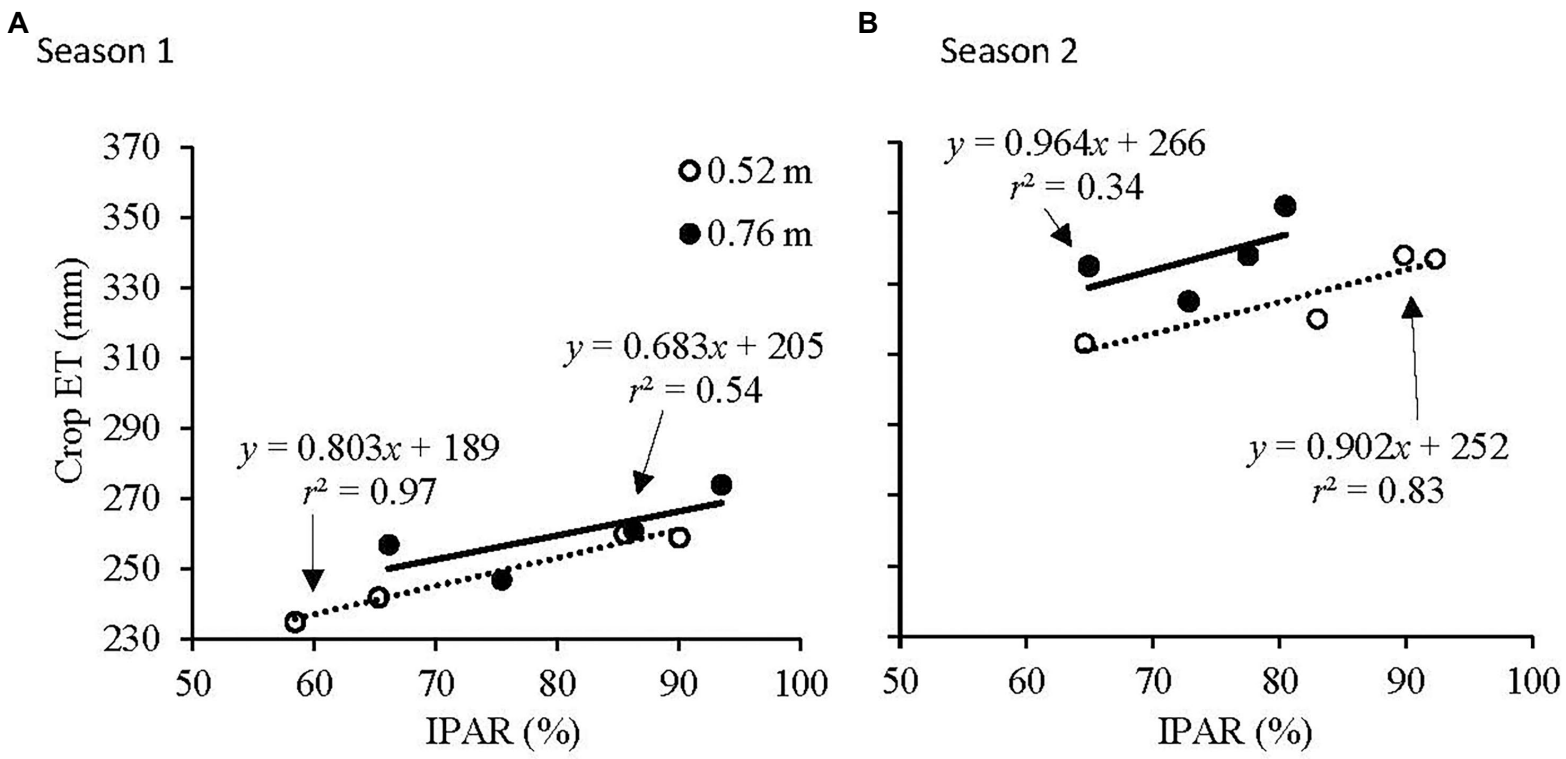
Relationship of seasonal crop evapotranspiration (crop ET) to intercepted photosynthetically active radiation (IPAR) at 0.52 and 0.76 m row spacings in **(A)** Season 1 and **(B)** Season 2.

Crop ET as a function of IPAR at the 0.52 and 0.76 m row spacings is illustrated in Figure 2. At the 0.52 m row spacing, there was a strong positive response of crop ET to IPAR in both seasons (*r*^2^ > 0.8; *p* < 0.05). At the 0.76 m row spacing, there was a positive response of crop ET to IPAR in both seasons, however, this response was weak (*r*^2^ > 0.3) in both seasons. Crop ET was greater for a given IPAR in the 0.76 m row spacing than in the 0.52 m row spacing, but both showed similar responses to increasing IPAR.

### Biomass Production

Total biomass at V6 was affected by the interaction of plant population and row spacing, without a season effect (*p* < 0.05). Total biomass at ≥50,000 plants ha^−1^ was greater than at lower plant populations at a similar row spacing due to lower interplant competition combined with adequate soil water levels during the first 4 weeks following planting (Table 3; *p* < 0.05). At 0.76 m row spacing, total biomass at 50,000 plants ha^−1^ was less than at 40,000 and 50,000 plants ha^−1^ (*p* < 0.05).

**Table 3:** Effect of row spacing and plant population on total biomass at the sixth-leaf collar (V6) development stage across season.

Row spacing (m)	Plant population (ha^−1^)	Total biomass (kg ha^−1^)
0.52	25,000	976^de^
	38,000	1,255^bcd^
	50,000	1,963^a^
	60,000	2,029^a^
0.76	20,000	868^d^
	30,000	1,065^cde^
	40,000	1,381^bc^
	50,000	1,465^b^

No common letter indicates a significant difference at level *p* < 0.05 in the ANOVA.

Total biomass at the VT, R3–R4, and R5–R6 development stages was affected by the interaction between row spacing and season (*p* < 0.05). There was, however, no response of total biomass at R5–R6 to plant population indicating the trade-off associated with increased plant population in semi-arid environments when available soil resources are insufficient to address the greater demand at higher densities. At VT in Season 1, total biomass was 14% greater at 0.52 m than at 0.76 m row spacing (*p* < 0.05), while no differences were observed in total biomass between row spacings at R3–R4 and R5–R6 development stages (Table 4; *p* > 0.05). In Season 2, total biomass was lower at VT, R3–R4, and R5–R6 development stages with both row spacings compared to Season 1 (*p* < 0.05). Total biomass at 0.76 m row spacing was 32, 30, and 33% more than at the 0.52 m row spacing at the VT, R3–R4, and R5–R6 development stages, respectively.

**Table 4:** Effect of season and row spacing on total biomass at the tasseling (VT) stage, the linear development phase of kernel filling (R3–R4), and physiological maturity (R5–R6) across all plant populations.

Season	Row spacing (m)	Total biomass (kg ha^−1^)		
		VT	R3–R4	R5–R6
Season 1	0.52	9,483^a^	10,476^a^	12,796^a^
	0.76	8,175^b^	9,887^a^	13,425^a^
Season 2	0.52	4,170^d^	5,290^c^	6,591^c^
	0.76	6,112^c^	7,501^b^	9,752^b^

No common letter indicates a significant difference at level *p* < 0.05 in the ANOVA.

**Figure 3:**
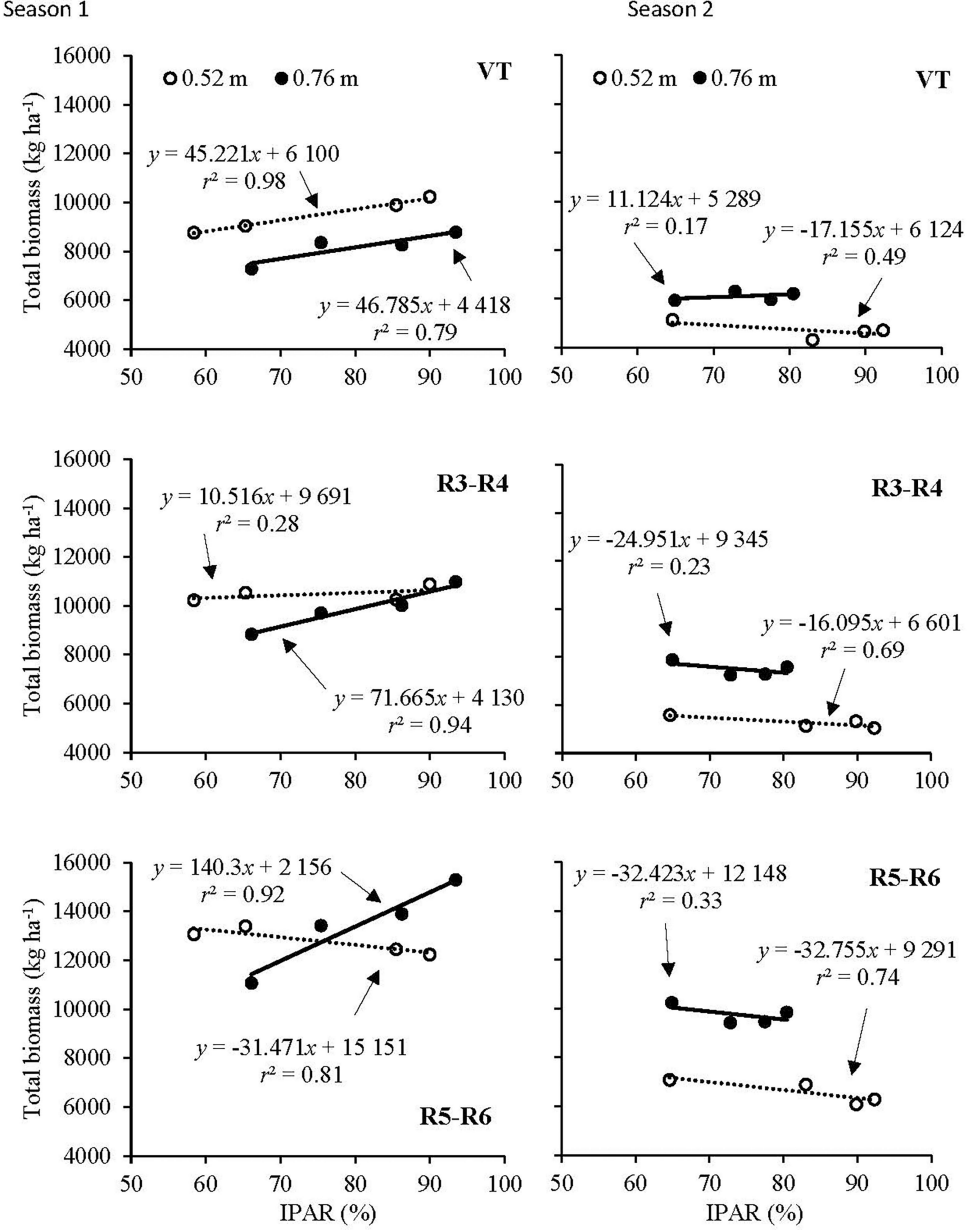
Relationship of total biomass to IPAR at the tasseling (VT) development stage, the linear development phase of kernel filling (R3–R4) and physiological maturity (R5–R6) development stage at the 0.52 and 0.76 m row spacings in Season 1 (left) and Season 2 (right).

Total biomass as a function of IPAR at 0.52 and 0.76 m row spacings is illustrated in Figure 3. In Season 1, there was a strong response of total biomass to IPAR at 0.76 m row spacing with increases of 468, 716, and 1,403 kg ha^−1^ for each additional 10% of IPAR at the VT, R3–R4, and R5–R6 stages, respectively. However, at the 0.52 m row spacing, the response of total biomass was positive at the VT stage (452 kg ha^−1^ per 10% increase in IPAR) but negative at the later growth stages.

Contrasting responses of total biomass to IPAR were observed in the drier Season 2. Total biomass at 0.52 m row spacing had a negative response to IPAR at VT, R3–R4, and R5–R6 (Figure 3). Although total biomass at 0.76 m row spacing had a positive response to IPAR at VT, the response was weak. At R3–R4 and R5–R6, a weak negative response of total biomass to IPAR was observed at 0.76 m.

### Grain Yield and Yield Components

Mean grain yield was considerably greater in Season 1 (8,119 kg ha^−1^) than in Season 2 (7,162 kg ha^−1^; Table 5). In Season 1, there were no yield differences between plant populations at the 0.52 m spacing or at populations less than 40,000 plants ha^−1^ at the 0.76 m spacing. However, the crop at 0.76 m spacing and 50,000 plants ha^−1^ yielded significantly more than all the other treatments. In Season 2 yield declined as population increased at the 0.52 m spacing, although the difference between yields at 50,000 and 60,000 plants ha^−1^ was not significant (*p* > 0.05). Yield was greater at the 0.76 m spacing and there were no yield differences between plant populations (*p* > 0.05).

**Table 5:** Effect of row spacing and plant population on grain yield, kernel weight, kernels per plant, grain yield per plant, and harvest index in Seasons 1 and 2.

Season	Row spacing (m)	Plant population (ha^−1^)	Grain yield (kg ha^−1^)	Kernel weight (g)	Kernels plant^−1^	Grain yield plant^−1^ (g)	Harvest index	WP_g_(kg mm^−1^)
Season 1	0.52	25,000	6,745^bc^	0.41^bc^	656^b^	270^b^	0.52^abc^	28.81^ab^
		38,000	6,804^bc^	0.41^bc^	439^de^	179^c^	0.51^abc^	28.08^abc^
		50,000	6,739^bc^	0.38^bc^	357^fgh^	135^d^	0.54^abc^	25.98^bc^
		60,000	6,366^c^	0.39^bc^	274^i^	106^de^	0.52^abc^	24.78^c^
	0.76	20,000	6,850^bc^	0.45^bc^	759^a^	342^a^	0.61^a^	26.68^bc^
		30,000	7,185^b^	0.44^bc^	535^c^	240^b^	0.53^ab^	29.11^ab^
		40,000	6,970^bc^	0.41^bc^	422^de^	174^c^	0.50^abc^	26.96^bc^
		50,000	8,580^a^	0.38^bc^	450^d^	172^c^	0.56^ab^	31.26^a^
Season 2	0.52	25,000	4,120^e^	0.41^bc^	404^def^	165^c^	0.58^ab^	13.20^de^
		38,000	3,001^f^	0.21^e^	379^efg^	76^f^	0.45^c^	9.39^ef^
		50,000	1,952^g^	0.12^f^	329^ghi^	41^g^	0.33^d^	5.79^fg^
		60,000	1,318^g^	0.09^g^	327^ghi^	22^h^	0.21^e^	3.97^g^
	0.76	20,000	5,280^d^	0.56^a^	418^def^	261^b^	0.52^abc^	15.79^d^
		30,000	4,685^de^	0.39^bc^	401^def^	155^c^	0.50^bc^	14.40^d^
		40,000	4,855^de^	0.34^cd^	356^efghi^	121^d^	0.51^abc^	14.35^d^
		50,000	5,100^d^	0.34^cd^	292^hi^	101^de^	0.51^abc^	14.47^d^

No common letter within the same column indicates a significant difference at level *p* < 0.05 in the ANOVA.

Kernel weight was similar across all treatments is Season 1 (Table 5; *p* > 0.05). In Season 2, kernel weight decreased with increasing plant population at 0.52 m row spacing (*p* < 0.05). Kernels per plant and grain yield per plant decreased with increasing plant population at both spacings and in both years although differences were not always significant (*p* < 0.05). Grain yield per plant decreased with increasing plant population at 0.52 and 0.76 m row spacing (Table 5; *p* < 0.05). In Season 1, grain yield per plant was higher at 0.76 m row spacing compared 0.52 m, with the opposite effect observed in Season 2 (*p* < 0.05). Harvest index remained constant across all treatments in Season 1, however, at 0.52 m row spacing in Season 2, harvest index decreased with increasing plant population (*p* < 0.05).

Neither crop ET nor WPb was affected by the plant population and row spacing treatments and were 24.58 and 51.85 kg mm^−1^ in Season 1 and 2 across treatments, respectively (data not shown). The response of WPg to the treatments was similar to the response of grain yield. In Season 1, WPg ranged from 24.8 to 31.3 kg mm^−1^, with differences between 25,000 and 60,000 plants ha^−1^ at 0.52 m row spacing and between 50,000 and 20,000 and 40,000 plants ha^−1^ at 0.76 m row spacing (Table 5; *p* < 0.05). In Season 2, WPg decreased (*p* < 0.05) with increasing plant population at 0.52 m row spacing, while WPg remained constant (*p* > 0.05) across plant population at 0.76 m row spacing. Treatment and seasonal effects on crop ET, WPb, and WPg during 2-week periods throughout the growing season were explored, however, no differences (*p* > 0.05) were found between treatments.

**Figure 4:**
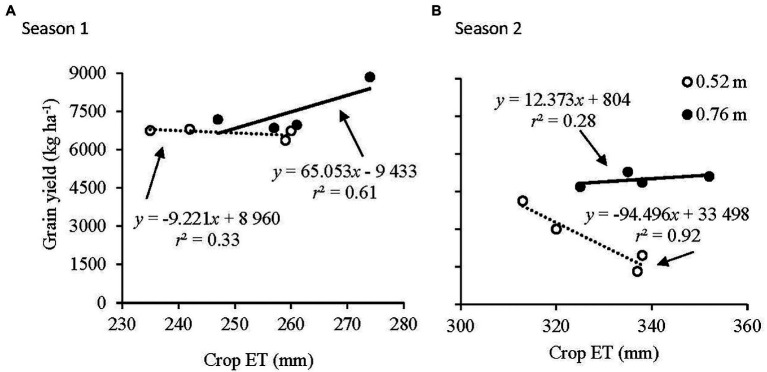
Relationship of grain yield to accumulated crop evapotranspiration (crop ET) at the 0.52 and 0.76 m row spacings in **(A)** Season 1 and **(B)** Season 2.

Grain yield as a function of crop ET at the 0.52 and 0.76 m row spacings is illustrated in Figure 4. In Season 1 at 0.52 m row spacing, a weak negative response of grain yield to crop ET was found, while a positive response in grain yield to crop ET was found at 0.76 m row spacing (Figure 4A). At 0.76 m row spacing, for each additional 10 mm of crop ET, maize grain yield increased by 651 kg ha^−1^. In Season 2, a strong negative response of grain yield to crop ET was found at 0.52 m row spacing, while a weak positive response of grain yield to crop ET was found at 0.76 m row spacing (Figure 4B). At 0.52 m row spacing, for each additional 10 mm of crop ET, grain yield decreased by 945 kg ha^−1^.

**Figure 5:**
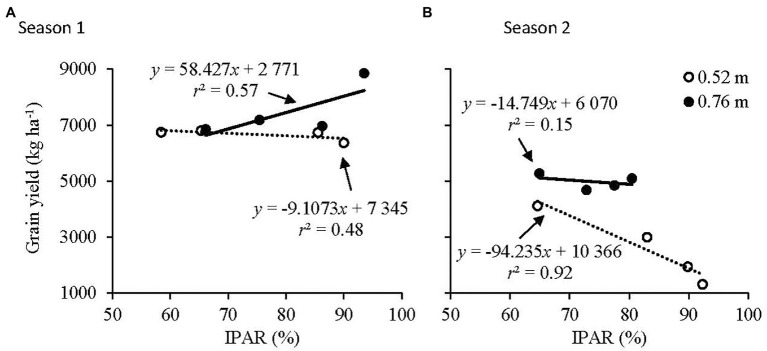
Relationship of grain yield to IPAR at the 0.52 and 0.76 m row spacings in **(A)** Season 1 and **(B)** Season 2.

Grain yield as a function of IPAR at 0.52 and 0.76 m row spacings is illustrated in Figure 5. In Season 1, a negative response of maize grain yield to IPAR was observed at 0.52 m row spacing, while the opposite was true at 0.76 m row spacing (Figure 5A). In Season 2, a strong negative response in grain yield to IPAR was observed at 0.52 m row spacing, while grain yield could not be explained by IPAR at 0.76 m row spacing (Figure 5B).

## Discussion

Intercepted photosynthetically active radiation is directly related to incident leaf canopy size and architecture (Flénet et al., 1996). Increased IPAR with increasing LAI is associated with higher plant populations (Fromme et al., 2019). Newly released maize hybrids underwent changes in aboveground morphology traits contributing to the success of greater plant populations (Duvick, 2005). Breeding efforts resulted in more vertical leaf growth above ears allowing more efficient sunlight interception and distribution throughout the leaf canopy (Mantilla-Perez and Salas Fernandez, 2017). Conserving soil moisture early in the season by developing less leaf area alongside improved root development may be beneficial in semi-arid environments with terminal droughts (Milander, 2015). In this study, when water-stress conditions occurred in Season 2, higher LAI and IPAR values were found at the narrower row spacing (0.52 m) compared to the 0.76 m row spacing when plant population were greater than 30,000 plants ha^−1^ (Table 2). The lower LAI and IPAR at the wider row spacing were advantageous later in the growing season when plants were in the reproductive stages, especially when soil water was limiting. Less vigorous vegetative growth and investment in biomass production early in the growing season (Table 3) may have resulted in a lower transpiration demand. This enabled the crop to utilize available soil water more effectively for grain production when rainfall arrived later in the season. At the higher plant populations, soil water levels became depleted as dry conditions persisted as the growing season advanced, resulting in greater competitiveness between developing plants. As a result, biomass production was similar between plant populations at physiological maturity (Table 4). Despite greater sunlight interception at the higher plant populations, greater biomass was not observed. Closed stomata may have inhibited photosynthesis when stressed conditions occurred, while senesced leaf area may have been included in the IPAR measurements. Allen (2012) found increased biomass and grain yield at lower plant populations when less than 300 mm of rainfall was received during the growing season, however, the plant populations investigated was much lower compared to the plant populations in our study.

Timing of water-stress influences the relationship between grain yield and yield components (Blumenthal et al., 2003; Milander, 2015). High rainfall at R3–R4 in Season 1 provided favorable conditions for kernel growth onward and may have reduced the competition for carbon-assimilates (Uribelarrea et al., 2008). The 41% decrease in kernel number per plant alongside no significant decrease in kernel weight from the lowest to highest plant population at 0.52 and 0.76 m row spacings counterbalanced the increase in the number of plants per ha. This led to no grain yield response to plant population in Season 1, except for the 50,000 plants ha^−1^ established at 0.76 m treatment. A similar decrease in kernel number per plant and ear length with increasing plant population was reported in below-average rainfall seasons (Cox and Cherney, 2012; Zhang et al., 2014).

In Season 2, hot and dry growth conditions prevailed for the majority of the latter vegetative development stages and early reproductive stages (Figure 1) which lowered yield potential by inhibiting photosynthesis, pollination, and carbohydrate translocation to kernels (Boyer, 1982; Schussler and Westgate, 1991). Soil water availability per plant was very low during the linear phase of kernel filling and ceased kernel filling. This slowdown in the crop’s life cycle was exacerbated with higher interplant competition exerted by the higher plant populations and narrower row spacing, resulting in low kernel weight and consequently poor grain yields despite increasing biomass production (Setter et al., 2001; Table 5). This explains the negative response of grain yield to crop ET in Season 2 at 0.52 m row spacing.

Cautious consideration must be given not only to plant population, but also the combination of plant population and row spacing in semi-arid environments. A maize grain yield of between 6,000 and 7,000 kg ha^−1^ is possible with plant populations of between 20,000 and 40,000 plants ha^−1^ irrespective of the row spacing. To achieve maize grain yields greater than 7,000 kg ha^−1^, it appears that a plant population in excess of 40,000 plants ha^−1^ is required at a row spacing of 0.76 m. The evidence of improved sunlight interception and ultimately higher biomass and maize grain yields at high plant populations and 0.76 m row spacing in seasons with more timely rainfall is clear. However, in semi-arid environments, deciding on a more optimal plant population and row spacing will ultimately be a compromise between obtaining high maize grain yield and minimizing the potential for stress-induced yield losses. In seasons with low rainfall, lower plant populations (<40,000 plants ha^−1^) will be associated with lower risk, but in seasons with adequate or plentiful rainfall a maize grain yield penalty could be expected (Birch et al., 2008). Although producers can use seasonal forecasts to adjust plant population at a given row spacing before planting, rainfall amount, and distribution throughout the particular season will ultimately determine if the approach is successful or not (Adisa et al., 2018, 2019). The higher seed costs associated with increased plant populations have a further impact on the decision-making process of producers (Lenssen et al., 2018), as economic losses increase when higher plant populations are established in dry seasons. Combining the economic (variable costs such as seed, labor, and fertilizer) and weather factors into a predictive model could produce a probability distribution of profit margin for each plant population management option.

## Conclusion

Vegetative growth, biomass production, and grain yield responded inconsistently to plant population and row spacing between seasons due to timing of rainfall in relation to growth stage. In seasons with low and poorly distributed rainfall, there was no clear indication of benefits in terms of biomass production, grain yield, or water productivity with increased plant population at both 0.52 and 0.76 m row spacings, although plant population treatments at 0.76 m row spacing outperformed plant population treatments at 0.52 m row spacing. This was mainly attributed to poorer growth during the vegetative development stages, enabling plants to utilize available soil resources more effectively later in the season. In low-rainfall seasons lower plant populations (<40,000 plants ha^−1^) will be associated with lower risk for crop failure, however, in seasons with plentiful rainfall a yield penalty could be expected. Although producers can use seasonal forecasts to adjust plant population at a given row spacing before planting, rainfall amount and distribution throughout the particular season will ultimately determine if the approach is successful or not. Developing prediction models by incorporating economic factors with weather-related factors such as rainfall amount and timing, and daily temperatures using long-term weather data (or generated weather for future scenarios) will serve as useful support tools. Producers and agronomists will be able to make better informed decisions when deciding on the optimal plant populations for a specific region and season.

## Data Availability Statement

The raw data supporting the conclusions of this article will be made available by the authors, without undue reservation.

## Author Contributions

SH and PS: conceptualization, methodology, data curation, and investigation. SH: draft preparation. PS: supervision, project administration, funding acquisition, and review and editing. All authors contributed to the article and approved the submitted version.

## Funding

This research was funded by The Maize Trust, AgriSETA, and the South African Society of Crop Production by providing respective PhD bursary programs.

## Conflict of Interest

The authors declare that the research was conducted in the absence of any commercial or financial relationships that could be construed as a potential conflict of interest.

## Publisher’s Note

All claims expressed in this article are solely those of the authors and do not necessarily represent those of their affiliated organizations, or those of the publisher, the editors and the reviewers. Any product that may be evaluated in this article, or claim that may be made by its manufacturer, is not guaranteed or endorsed by the publisher.
