# Supplementary material for: Plant Population and Row Spacing Affects Growth and Yield of Rainfed Maize in Semi-arid Environments
Source: Front Plant Sci. 2022 Jun 8;13:761121. doi: 10.3389/fpls.2022.761121 (PMC9214209; doi:10.3389/fpls.2022.761121)
Supplement: Supplementary file 1 [file Table_1.docx]

**Table S1.** Comparison of soil water content (mm) in the 1.2 m soil profile between various plant population and row spacing configurations at different stages throughout the growing season in Season 1 and 2.

| Season | Row spacing (m) | Plant population (plants ha^-1^) | Soil water content at various days after emergence (mm) | | | | | | | |
| --- | --- | --- | --- | --- | --- | --- | --- | --- | --- | --- |
|  |  |  | 7 | 21 | 35 | 49 | 63 | 83 | 103 | 120 |
| Season 1 | 0.52 | 25 000 | 117 | 122 | 114 | 102 | 83 | 72 | 101 | 104 |
|  |  | 38 000 | 120 | 123 | 112 | 98 | 75 | 65 | 105 | 107 |
|  |  | 50 000 | 121 | 123 | 107 | 90 | 70 | 64 | 100 | 102 |
|  |  | 60 000 | 122 | 120 | 104 | 89 | 75 | 67 | 101 | 102 |
|  | 0.76 | 20 000 | 120 | 122 | 104 | 88 | 71 | 63 | 98 | 106 |
|  |  | 30 000 | 117 | 126 | 108 | 92 | 75 | 63 | 101 | 106 |
|  |  | 40 000 | 118 | 115 | 90 | 80 | 67 | 58 | 95 | 97 |
|  |  | 50 000 | 119 | 116 | 91 | 78 | 68 | 59 | 98 | 102 |
| Season 2 | 0.52 | 25 000 | 106 | 91 | 77 | 69 | 83 | 56 | 101 | 104 |
|  |  | 38 000 | 110 | 92 | 76 | 66 | 75 | 51 | 105 | 107 |
|  |  | 50 000 | 113 | 92 | 73 | 61 | 70 | 48 | 100 | 102 |
|  |  | 60 000 | 112 | 90 | 70 | 60 | 75 | 51 | 101 | 102 |
|  | 0.76 | 20 000 | 110 | 91 | 71 | 61 | 71 | 48 | 98 | 104 |
|  |  | 30 000 | 114 | 94 | 73 | 63 | 75 | 51 | 101 | 104 |
|  |  | 40 000 | 114 | 86 | 61 | 54 | 67 | 45 | 95 | 95 |
|  |  | 50 000 | 115 | 87 | 62 | 52 | 68 | 46 | 98 | 101 |
